# Secret Remedies of the Past

**Published:** 1920-10-23

**Authors:** 


### SECRET REMEDIES OF THE PAST.

### BY A CORRESPONDENT.

Not long ago Parliament appointed a Select Committee "to inquire into the question of the Sale of Patent and Proprietary Medicines and Medical Preparations and Appliances and Advertisements relating thereto, and to report what Amendments, if any, in the law are necessary or desirable." This Committee is well representative of the interests concerned, medicine, pharmacy, and the Press being personified in its members. Public interest in the foregoing statement is possibly very slight, and it is doubtful whether the conclusions of the Committee will receive much attention, excepting from those whose livelihood is closely affected.

### Early Monopolies and Restrictions.

Upon what does the successful secret medicine business depend most? Is it the credulity of the consumer, the astuteness of the proprietor, or the intrinsic value of the article purveyed? I leave the question unanswered, well knowing that whatever answer I give will be unsatisfactory to some, and desiring here, above all things, to preserve your goodwill. Sufficient to say that the industry is a very old one, and that Parliamentary interference is no innovation. In 1628 a monopoly of the sale of Orvietan in the Papal States was granted, and the fine for disregarding the monopolists' rights was 1,000 ducats and excommunication. This Orvietan was a variety of medicament of a very ancient history, older than the Christian faith, which was called Mithradate or Venice Treacle. It was no simple concoction; it always had some sixty and more ingredients, including viper's flesh, and was, of course, recommended for innumerable complaints, amongst which it was stated to be an antidote to poisoning. This was a useful attribute in those days. In England the granting of these monopolies was a favourite method adopted by rulers to obtain money, and the abuse of these powers resulted in the passing of the Statute of Monopolies in 1624, which placed the power in the hands of Parliament and restricted the privilege to a period of fourteen years. At the present day so-called patent medicines are not always patented, but have only the name registered as a trade-mark, and thus the secret is preserved inviolate.

### The First Patented Medicine.

The first patent granted for a compound medicine was in 1711 to one Timothy Byfield for his *Sal oleosum volatile*. Of this no particulars of manufacture or ingredients are given. Dr. James's Fever Powder (1747), John Hooper's Pills (1743), Frier's Drops (1777) were amongst the early entrants to list of patents; the last was possibly the forerunner of the present-day Friar's Balsaam. As an example of a long-lived proprietary article, perhaps Singleton's Golden Eye Ointment is remarkable. It is claimed that it was first made in 1596 and has been continuously sold and used until to-day. Parliamentary interference took a form in 1739 which is not likely to be repeated by the present inquiry. There was passed that year "An Act for providing a reward to Joanna Stephens upon a proper discovery to be made by her for the use of the publick of the Medicines prepared by her for the Cure of the Stone." The lady, an estimable widow, had inherited the recipes from her husband. Society had taken to the remedy, and many cures were reported.

### Parliament Votes £5,000 for Secret Remedy.

In the *Gentleman's Magazine*, in 1738, a movement was started to buy the formula from the lady, who had indicated her willingness to accept £5,000 for the secret. Several bishops and responsible persons testified to the usefulness of the remedy, and in a few days £500 was forthcoming. However, after one year there was only £1,350 in the fund, so Parliament was approached and the complete sum was voted and duly paid to Mrs. Stephens. The recipes were published in the *London Gazette* of June 19, 1739, and were extremely disappointing. The remedy took the form of a powder, a decoction and pills. The powder was of egg-shells and garden snails, each calcined and then mixed. The decoction was of soap and about five herbs. The pills were of burnt snails mixed with certain seeds. The purchase and publication was so far beneficial as it directed attention to the use of alkaline medicines for the disease, but no cures were effected by the treatment. According to Dr. Pereira, the stone was found after death in each of the four test cases.

### Royal and Parliamentary Patronage.

Joshua Ward, who was proprietor of Ward's Paste and several other secrets, was one of the most successful patent-medicine vendors. He was able to obtain the patronage of George II. by treating that ruler's dislocated thumb, and when in 1748 a Bill was introduced into Parliament to regulate the practice of medicine, there was a special clause exempting Ward by name from its penalties. It is related that Queen Charlotte, asking a courtier whether it was true that Ward's medicines had made a man mad, received the reply, "Yes, Madam, Mead." Dr. Mead was the King's physician and a famous regular practitioner. One of the most famous of the extensive list of remedies was Warburg's Tincture, which was invented by an Austrian doctor of that name early in the nineteenth century. The success of this tincture in India for treating fevers of every degree was a matter of great interest, and finally the original formula was entrusted by Dr. Warburg to the medical profession. It contained aloes, rhubarb, myrrh, gentian, saffron, and quinine amongst a multitude of other ingredients, which also included *Confectio Damocratis*. This last confection was the survivor of the ancient concoction referred to earlier as Mithradate. Dr. Warburg was reduced to great poverty after his formula was made public, and a grant in aid was made by the India Office. Later a small pension for the inventor was raised by subscription.

